# Cutaneous and non‐cutaneous diseases due to *Mycoplasma pneumoniae* in children

**DOI:** 10.1111/ddg.70052

**Published:** 2026-03-05

**Authors:** Hanna Lindemann, Maximilian David Mauritz, Victoria Rehmann, Michael Paulussen, Jorge Frank, Malik Aydin

**Affiliations:** ^1^ Department of Dermatology and Allergology University Hospital Basel Basel Switzerland; ^2^ Chair of Pediatrics, Children's Hospital, Vestische Kinder‐ und Jugendklinik Datteln, Witten/Herdecke University Datteln Germany; ^3^ Department of Dermatology, Venereology, and Allergology University Medical Center Göttingen Göttingen Germany; ^4^ Laboratory of Translational Medicine and Pediatric Infectious Diseases, Faculty of Medicine Department of Human Medicine Witten/Herdecke University Witten Germany; ^5^ Virology and Microbiology, Center for Biomedical Education and Research (ZBAF) Department Human Medicine Faculty of Health, Witten/Herdecke University Witten Germany; ^6^ Institute for Medical Laboratory Diagnostics Center for Clinical and Translational Research Helios University Hospital Wuppertal Witten/Herdecke University Wuppertal Germany

**Keywords:** erythema multiforme, erythema nodosum, Mycoplasma‐induced rash and mucositis, Mycoplasma pneumoniae, Stevens‐Johnson syndrome, toxic epidermal necrolysis

## Abstract

*Mycoplasma pneumoniae* (MP) is a common pathogen responsible for diverse infections in children and adolescents, primarily affecting the respiratory tract. Besides causing atypical pneumonia, MP can also lead to extrapulmonary manifestations, including mucocutaneous, hematological, neurological, cardiac, and gastrointestinal symptoms. These extrapulmonary manifestations are often overlooked, complicating diagnosis and management. Here, we delineate the spectrum of cutaneous and non‐cutaneous diseases caused by MP in children, focusing on the pathophysiology, clinical features, and treatment of mucocutaneous manifestations.

## INTRODUCTION

*Mycoplasma pneumoniae* (MP) is a small pleomorphic bacterium of the Mollicutes class. It is primarily transmitted through respiratory droplets, reflecting a common cause of community‐acquired pneumonia, particularly in school‐age children.^1^ The peak incidence is in late summer and fall, with epidemics recurring every three to seven years.^1^ After the end of the COVID‐19 measures, an increase in infections was notable, highlighting the importance of this pathogen.^2^

Following transmission, MP adheres to the respiratory epithelium, enabling colonization. Asymptomatic carriage may persist for weeks to months, even under antimicrobial therapy.^3^ After colonization, MP releases cytotoxins that can affect host tissues and activate both local and systemic inflammatory responses. This process leads to a wide range of disease manifestations, with the majority being due to indirect immune‐mediated effects.^1^, ^4^

## DIAGNOSIS

The diagnosis is based on clinical evaluation and exposure history, supported by laboratory testing. Species‐specific polymerase chain reaction (PCR) assays offer high sensitivity but cannot reliably differentiate between colonization and active infection.^3^, ^5^ Serological testing is commonly utilized, with IgM antibodies usually being released at early phases, followed by detection of IgG antibodies three to four weeks post‐infection.^5^ Identification of MP‐specific IgM secreting cells in peripheral blood is the strongest indication of acute disease. Nevertheless, this method has not yet been implemented in clinical practice due to the self‐limiting disease course.^6^ Moreover, cultures are rarely performed due to difficult and slow growth of the bacteria.

Depending on the clinical presentation, the diagnosis can also be supported by imaging methods, including chest X‐rays, to confirm suspected pneumonia.^7^, ^8^

## CLINICAL MANIFESTATIONS

### Non‐cutaneous diseases

Pneumonia is the most common clinical manifestation of MP in school‐age children. Following an unproductive cough, patients usually suffer from low‐grade fever, headache, and malaise.^7^ Radiologic findings are variable and mostly non‐specific, including uni‐ or bilateral lower lobe or perihilar bronchopneumonia with reticulonodular opacity.^8^ Extrapulmonary manifestations may also occur. In addition, IgM antibodies to the erythrocyte membranes may produce a cold‐agglutinin response in almost 50% of patients.^9^ However, a clinically significant hemolysis may affect patients with chronic diseases, including sickle cell anemia. Furthermore, central nervous system manifestations, including meningoencephalitis and acute disseminated encephalomyelitis, may occur in approximately 5% of hospitalized pediatric patients. Gastrointestinal symptoms, particularly abdominal pain, vomiting, and diarrhea can also occur.^10^ Rheumatologic, cardiac, and renal disease are only rarely observed among pediatric patients.^10^

While respiratory tract infections are the hallmark of MP infection, the lipid‐associated membrane proteins are also recognized for their potential to act as superantigens.^11^ Systemic immune response correlates with the severity of respiratory disease and can lead to different extrapulmonary manifestations.^12^, ^13^

Biologically, MP has a unique structure that lacks a true cell wall, which presents a physiological resistance against β‐lactam antibiotics. In contrast, macrolides, tetracyclines, and fluoroquinolones present the antibiotics of choice. However, fluoroquinolones are not primarily indicated in children due to the equal effectiveness of other agents and the risk of irreversible cartilage damage. Since most respiratory infections caused by MP are self‐limiting, a “watchful waiting” procedure is the most adequate option for non‐severe disease, and antibiotic treatment should be reserved for patients with severe or prolonged course.^1^ Future studies must demonstrate whether treatment with macrolides affects the course of community‐acquired pneumonia caused by MP at all.^10^ Table 1 summarizes the most prevalent non‐cutaneous manifestations associated with MP in children.

**TABLE 1:** The most prevalent non‐cutaneous diseases and manifestations in children associated with *Mycoplasma pneumoniae* infections.^9^, ^10^, ^11^, ^12^, ^13^, ^14^

Disease(s)	Organ involvement/manifestations	Clinical symptoms/features
*Mycoplasma pneumonia*	Lower respiratory tract infection	Fever; unproductive cough; fatigue; dyspnea and/or headache
Pharyngitis Asthma	Other respiratory manifestations	Sore throat; coryza; headache; ear pain; prolonged cough or wheezing
Secondary cold agglutinin syndrome	Hematologic disease	Clinically not relevant hemolysis or severe hemolysis in risk patients with e.g., sickle cell anemia
Meningoencephalitis Acute disseminated encephalomyelitis Transverse myelitis Cerebellar ataxia Guillain‐Barré syndrome Cerebral/cerebellar infarction Peripheral neuropathy Cranial nerve palsies	Central nervous system manifestations	Neurological symptoms corresponding to the respective manifestations; lymphocytic pleocytosis; elevated protein levels and normal glucose levels
Splenomegaly Intussusception Hepatomegaly Pancreatitis	Gastrointestinal manifestations	Abdominal pain; vomiting; diarrhea
Septic or reactive arthritis or arthralgia Rhabdomyolysis	Rheumatologic and musculoskeletal manifestations	Joint or muscle pain and/or swelling
Myocarditis Pericardial effusion Intravascular thrombi Kawasaki disease Heart failure	Cardiovascular manifestations	Tachy‐/Bradycardia; heart failure
Glomerulonephritis secondary to immune complex deposition	Renal manifestations	Acute nephritis; nephrotic syndrome or acute renal failure and proteinuria

### Cutaneous diseases

Cutaneous symptoms occur in 10% to 25% of MP infections, with children being more affected than adults.^7^ Skin manifestations can be associated with both active and asymptomatic infections, including non‐specific maculopapular exanthema, Mycoplasma‐induced rash and mucositis (MIRM), erythema nodosum (EN), urticaria, erythema multiforme (EM), and Stevens‐Johnson syndrome (SJS). Rare disorders include pityriasis rosea, toxic epidermal necrolysis (TEN), immune complex vasculitis, thrombotic thrombocytopenic purpura (TTP), subcorneal pustular dermatosis (Sneddon‐Wilkinson syndrome), Sweet syndrome, Raynaud phenomenon, Reiter syndrome, urticarial vasculitis, and Gianotti‐Crosti syndrome (Table 2).^4^, ^6^, ^10^, ^14^

**TABLE 2:** The most prevalent skin diseases in children associated with *Mycoplasma pneumoniae* infections.^7^ ^˒^ ^1^ ^6^ ^˒^ ^1^ ^7^

Disease	Frequency of all *M. pneumoniae*‐related cases	Skin manifestation(s)	Distribution	Mucosal involvement	Systemic involvement
Non‐specific maculopapular exanthema	8–33%	Maculopapular rash	Trunk and extremities ± palms and soles	Usually absent Less common; only described in individual cases	Usually mild or absent
Mycoplasma‐induced rash and mucositis	13 cases	Minimal or absent polymorphic rash Typical vesiculobullous or atypical target lesions	Predominance of mucosal involvement Less than 10% BSA* detachment	Severe; ≥ 2 mucosal sites (e.g., oral, ocular, anogenital, nasal)	Usually moderate; fever, malaise
Erythema nodosum	8%	Painful erythematous nodules	Most common on the shins Less common on the forearms and thighs	Absent	Usually mild; fever, arthralgia, malaise
Urticaria	7%	Annular plaques ± swelling of face, hands or feet (angioedema)	Widespread Individual lesions are transient (< 24 h)	Absent	Usually mild or absent
Erythema multiforme minus	3,6–7%	Typical target lesions ± papular atypical target lesions	Extremities	Absent or mild	Usually mild or absent
Erythema multiforme majus		Typical target lesions ± papular atypical target lesions ± bullous lesions	Extremities and face	Severe	Usually mild; fever, arthralgia
Stevens‐Johnson syndrome	1–%	Typical target lesions with epidermal detachment and erosions Bullous lesions	Isolated lesions with confluence on the trunk and face Less than 10% body surface area detachment	Severe	Usually severe; fever, lymphadenopathy, hepatitis, cytopenia

### Non‐specific maculopapular exanthema

The most common MP‐associated cutaneous manifestation is a non‐specific maculopapular exanthema. In a prospective study of 152 children with community‐acquired pneumonia, 22.7% of those with PCR‐confirmed MP and specific IgM‐secreting cells developed mucocutaneous lesions.^4^ Pruritic rash is often seen in early disease stage, typically manifesting with small red macules or flat plaques that may involve large body areas, including the palms, soles, and mucous membranes. The exanthema is usually self‐limiting within 2 to 22 days and mostly responds well to symptomatic treatment with antihistamines and topical corticosteroids.^4^

Differentiating between MP‐induced exanthema and drug‐induced eruptions is crucial. A retrospective analysis of 81 patients with serologically confirmed MP infection showed that 18.5% developed skin rash, with 72% having received two or more antibiotics, predominantly ampicillin or other penicillin, suggesting potential drug‐related causes.^15^

### Mycoplasma‐induced rash and mucositis

In 2015, Canavan et al. firstly described MIRM as a distinct disorder primarily affecting preadolescent boys (mean age 11.9 years).^16^ It comprises mucositis involving at least two sites (oral, ocular and/or genital) with limited skin involvement (< 10% of the body surface), often presenting with few vesiculobullous or target lesions. Systemic symptoms may include fever, cough, and abnormal lung findings or imaging. MP infection can be confirmed through elevated IgM antibody levels or PCR.^16^

The delineation of MIRM as an entity different from EM and SJS has caused an ongoing debate. Clinically, MIRM shares overlapping features with EM major and SJS, including mucosal involvement and target lesions. Histopathology has not demonstrated convincing differences between these conditions, further complicating their distinction.^16^, ^17^

Mucosal affection may require evaluation by ophthalmology, otolaryngology, gastroenterology, or urology. While there are no treatment guidelines, early antibiotics, and supportive care, including pain control, mucosal care, and fluid management, can be helpful. Effective management of painful mucosal lesions is imperative to prevent secondary infections. Severe mucosal involvement may benefit from intravenous immunoglobulins, and severe skin involvement may require involvement of specialized burn centers.^16^, ^17^

Recent findings suggest that similar mucocutaneous manifestations can occur with other infectious agents, prompting the use of the broader term “reactive infectious mucocutaneous eruption” (RIME).^18^

### Erythema nodosum

EN is a rare cutaneous manifestation in children, characterized by painful red nodules, typically on the lower extremities.

One study identified MP infection by serological testing in three out of 35 children with EN (8.6%). Interestingly, one patient was asymptomatic.^19^ A second study detected MP in four of 39 pediatric patients with EN by positive IgM serology, most of them being symptomatic. In two cases, dual infection involving MP and another pathogen was demonstrated.^20^

Given the relatively low detection rate, MP testing in children with EN is not routinely recommended, particularly not in the absence of respiratory symptoms. However, targeted testing may be appropriate during MP outbreaks or in the presence of concurrent respiratory symptoms. While EN usually resolves spontaneously within few weeks, successful treatment with macrolides has been shown, probably reducing recurrence.^4^, ^10^

### Urticaria

MP‐associated urticaria is primarily seen in children. A study comprising 65 children with urticaria revealed MP IgM and/or a positive rapid cold agglutination test in 21 (32%) of them.^20^ Although clinical manifestation alone cannot confirm causality, MP testing is advisable in children with urticaria and concurrent respiratory symptoms. In such patients, antibiotic treatment may help reduce the duration of both respiratory symptoms and urticaria.^4^, ^21^

### Erythema multiforme, Stevens‐Johnson syndrome and toxic epidermal necrolysis

EM, SJS, and TEN reflect a spectrum of mucocutaneous disorders that are closely related but differ regarding clinical manifestation and disease severity.

**FIGURE 1:**
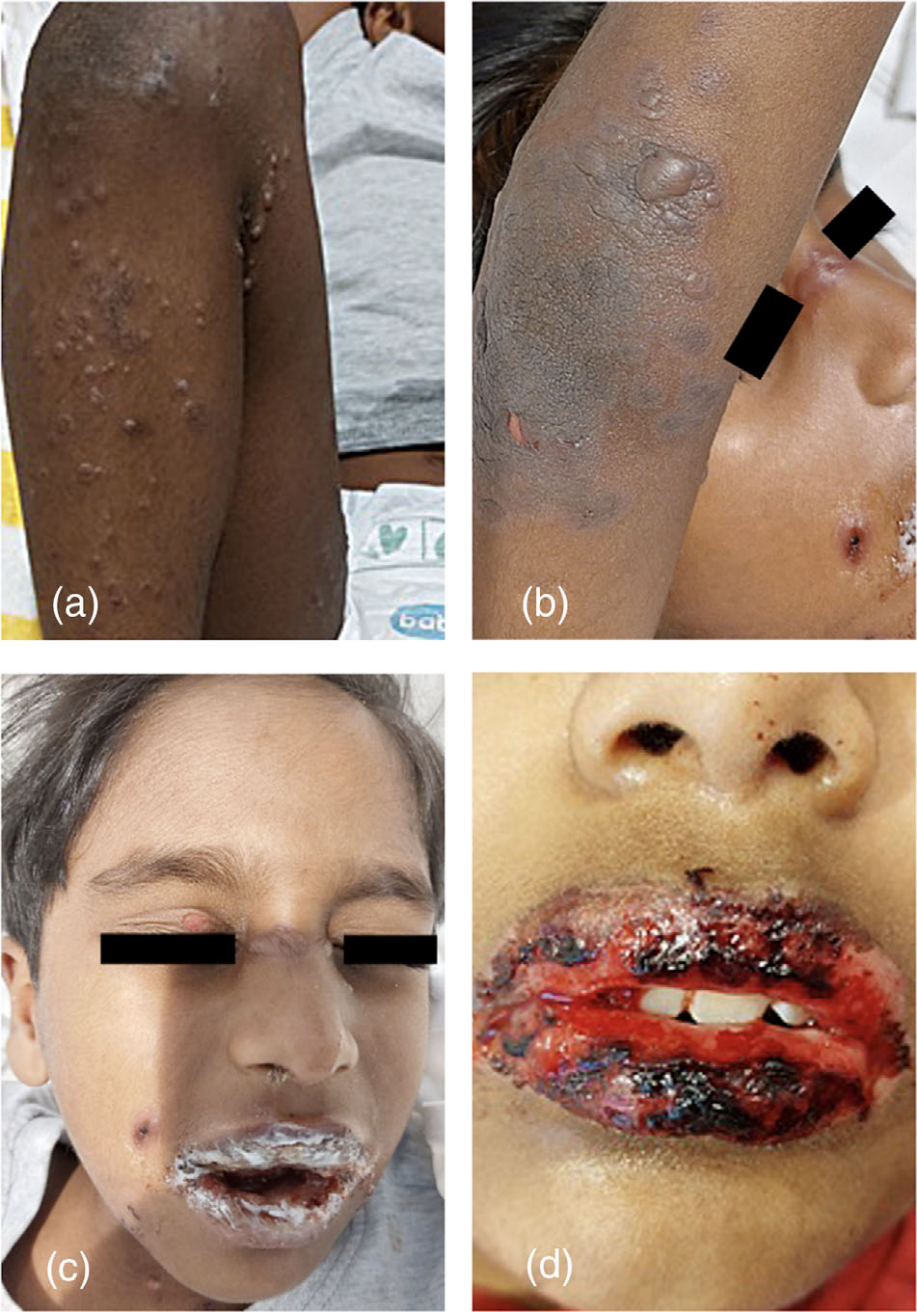
5‐year‐old boy with (a) solitary to aggregated papules, vesicles, and blisters on partly erythematous and partly unchanged skin with partial cockade (target lesion) formation on the right lower extremity; (b) solitary vesicles, blisters, and erosions on erythematous skin with partial cockade formation on the right upper extremity; (c) confluent and partially hemorrhagic erosions and crusts on the lips, crusts at the right nasal entrance, perioral papules and erosions on a partly erythematous background, as well as facial milia; (d) 10‐year‐old boy with confluent hemorrhagic erosions and crusts on the lips (Permission for reproduction of photographic material from Lindemann H et al., Kinderärztliche Praxis 96, 29–33, 2025, was obtained from MedTriX, Inc., Unter den Eichen 5, 65195 Wiesbaden, Germany. Copyright for photographic material is owned by Dermanostic, Inc., Merscheider Strasse 1, 42699 Solingen, Germany).

EM is classified into minor (without mucosal involvement) and major (with mucosal involvement) forms. A recent study identified MP as the second most frequent trigger of EM in children, accounting for approximately 7% of cases confirmed by IgM, IgG serology, and clinical presentation.^22^ Clinically, multiple target lesions on the extremities, including the palms and soles, can be also seen with minimal systemic involvement beyond mild fever or malaise. In some patients, however, mucosal involvement may occur, requiring hospitalization (Figure 1).^14^, ^22^

In contrast, SJS presents with confluent, atypical target lesions predominantly on the trunk with obligatory mucosal involvement. MP is the predominant infectious agent in SJS, accounting for approximately 22% of affected children.^23^

Clinical overlap between EM and SJS is well documented, whereas progression from EM to TEN has not been demonstrated yet.^24^

Currently, there are no standardized treatment protocols for MP‐induced EM, SJS, or TEN. However, early initiation of systemic corticosteroids and antibiotics may improve disease course. Supportive care, as outlined for MIRM, is essential and may require interdisciplinary collaboration.^24^

## CONCLUSIONS

MP infections can manifest with a broad range of non‐respiratory symptoms and disease manifestations, in particular in pediatric patients. Through local inflammatory cytokines, the membrane of MP may act as a super‐antigen, thereby resulting in immune system dysregulation.

Detection of non‐respiratory manifestations associated with MP is crucial for early diagnosis. In children, rapid identification of infection is important as it may influence treatment and prevent potential complications. However, distinguishing between active infection and asymptomatic colonization remains a diagnostic challenge, as MP can also persist in the upper respiratory tract of healthy individuals. This underscores the requirement for cautious interpretation of diagnostic results to avoid unnecessary antibiotic use.

## CONFLICT OF INTEREST STATEMENT

H.L. has received honoraria for oral presentations from Blueprint Medicines, Novartis, and OrPha Swiss GmbH. J.F. has received financial support for the organization of scientific meetings, symposia, and conference travel, as well as honoraria for consulting and oral presentations from AbbVie, Alnylam Pharmaceuticals, Bristol‐Myers Squibb, Clinuvel Pharmaceuticals, Genzyme, La Roche‐Posay, Lilly Deutschland GmbH, MedKomAkademie GmbH, MSD, Novartis, Orphan Europe, and Shire. He is a senior consultant at Dermanostic, Inc. and an associate partner at medi‐login, Inc. M.D.M., V.R., M.P., and M.A. declare no conflict of interest. The authors declare that economic interests had no influence on the preparation of the manuscript.
